# Quality of Wild Passion Fruit at Different Ripening Stages Under Irrigated and Rainfed Cultivation Systems

**DOI:** 10.3390/plants14142147

**Published:** 2025-07-11

**Authors:** Giuliana Naiara Barros Sales, Marília Hortência Batista Silva Rodrigues, Toshik Iarley da Silva, Rodolfo Rodrigo de Almeida Lacerda, Brencarla Lima Medeiros, Larissa Felix Macedo, Thiago Jardelino Dias, Walter Esfrain Pereira, Fabio Gelape Faleiro, Ivislanne de Sousa Queiroga Lacerda, Franciscleudo Bezerra da Costa

**Affiliations:** 1 Program of Postgraduate in Agronomy, Universidade Federal da Paraíba, Areia 58397-000, PB, Brazil; giulianasales@outlook.com (G.N.B.S.); thiagojardelinodias@gmail.com (T.J.D.); walterufpb@yahoo.com.br (W.E.P.); 2 Program of Postgraduate in Tropical Horticulture, Universidade Federal de Campina Grande, Pombal 58429-900, PB, Brazil; rodolfo-lacerda@hotmail.com (R.R.d.A.L.); mbrencarla@gmail.com (B.L.M.); larissafelixmcd@gmail.com (L.F.M.); franciscleudo.bezerra@professor.ufcg.edu.br (F.B.d.C.); 3 Center for Agrarian, Environmental, and Biological Sciences, Universidade Federal do Recôncavo da Bahia, Cruz das Almas 44380-000, BA, Brazil; 4 Empresa Brasileira de Agropecuária e Abastecimento Cerrados, Brasília 70000-000, DF, Brazil; fabio.faleiro@embrapa.br; 5 Paraíba State Department of Education, Pombal 58840-000, PB, Brazil; ivislannequeiroga@gmail.com

**Keywords:** BRS Sertão Forte, bioactive compounds, water stress, wild passion fruit, physiological maturity

## Abstract

*Passiflora cincinnata* (Mast), native to the Brazilian semi-arid region, produces exotic fruits even under low water availability. However, its green coloration at ripening complicates optimal harvesting, impacting post-harvest fruit quality. Therefore, this study aimed to evaluate the influence of cultivation systems (irrigated and rainfed) and different ripening stages on the physical and post-harvest characteristics of wild passion fruit during the second production cycle. The experiment was conducted using a randomized block design in a 2 × 4 factorial scheme, corresponding to two cultivation systems (irrigated and rainfed) and four fruit ripening stages (60, 80, 100, and 120 days after anthesis—DAA), with five replications. The fruit pulps were analyzed for physicochemical characterization and bioactive compounds. The physical and chemical characteristics of wild passion fruit were influenced by ripening stages and the irrigation system. The rainfed system decreased the total fruit mass by 15.50% compared to the irrigated cultivation. Additionally, the rainfed cultivation reduced the fruit color index by 14.82% and altered the respiratory pattern, causing a linear decrease of 73.37% in the respiration rate during ripening, in contrast to the behavior observed in the irrigated system, which reached an estimated minimum rate of 33.74 mg CO_2_ kg^−1^ h^−1^ at 110 days after anthesis.

## 1. Introduction

Fruit quality is an essential aspect for the success of agricultural production, as it directly affects market acceptance, consumption, and industrialization potential [1]. In the case of wild passion fruit (*Passiflora cincinnata* Mast.), a species native to the Brazilian Cerrado and Caatinga biomes, it produces fruits of a characteristic green color and is popularly known as Caatinga passion fruit [2,3].

In this context, efforts have been made to develop cultivars adapted to semi-arid regions, such as BRS Sertão Forte, obtained from a cross between two populations (CBAF2334 and CPEF2220) selected in the Brazilian semi-arid region. Under conditions in the state of Pernambuco and the Cerrado of the Central Plateau, this cultivar yields between 18 and 30 t ha^−1^ year^−1^, depending on the management practices adopted. It differs from the cultivated species Passiflora edulis by its greater tolerance to drought and poor soils [4]. Most of the fruits currently available are sourced through extractivism, which leads to high variability in yield and quality. This species has garnered increasing interest due to its resilience and adaptability to adverse conditions, such as low water availability and chemically and physically restrictive soils, which are often found in the Brazilian semi-arid region [2,3]. Additionally, it exhibits natural resistance to the cowpea aphid-borne mosaic virus (CABMV) [5,6].

Fruit quality is influenced by several factors, with ripening stages and water management systems being two key elements in determining physical, chemical, and sensory attributes [7]. During the ripening process, significant metabolic transformations occur, affecting properties such as fruit weight, firmness, coloration, soluble solids content (°Brix), titratable acidity, and the sugar/acid ratio, which together define fruit acceptability for different purposes [8,9]. Fruits harvested prematurely may exhibit less pleasant flavor and lower sugar content [10,11], while those harvested at advanced ripening stages may have a reduced shelf life due to firmness loss, transpiration, and increased susceptibility to pathogen attacks, e.g., *Solanum lycopersicum* [12,13].

In *Passiflora edulis* fruits, physiological ripening is characterized by a change in peel coloration and the natural abscission of the fruit from the plant. However, these changes do not occur in *Passiflora cincinnata* fruits, making the optimal harvest time more challenging to identify [14]. In sour passion fruit (*P. edulis*), the relationship between soluble solids and titratable acidity (SS/TA) tends to decrease during periods of milder temperatures [15]. Additionally, climatic conditions such as lower precipitation and cooler temperatures reduce the levels of reducing sugars and the SS/TA ratio compared to periods with higher temperatures [16]. Fruits harvested between May and September, under conditions of milder temperatures and lower precipitation, exhibit higher acidity, dry matter, total soluble solids, and SS/TA ratio compared to fruits harvested from October to December [17]. Therefore, determining the ideal ripening stage of wild passion fruit is essential to optimize both fruit quality and utilization.

Furthermore, the cultivation system directly influences fruit development and quality. In irrigated cultivation, plants receive a continuous and controlled water supply, which promotes uniform fruit development and can result in higher productivity [7]. On the other hand, rainfed cultivation, widely used in semi-arid regions, relies exclusively on rainfall and is therefore subject to water stress, which can negatively affect plant growth and final fruit quality, e.g., in *Solanum tuberosum* [18] and *Solanum lycopersicum* L. [19].

Water and nutrient deficits are among the primary causes of low productivity in commercial passion fruit (*Passiflora*) cultivation [20]. Water scarcity triggers a series of signaling cascades in plants, involving osmotic sensors present in the membrane that promote the accumulation of Ca^2+^ in the cytosol [21]. This process activates a phosphorylation cascade, resulting in changes in gene expression and the synthesis of the abscisic acid (ABA) hormone, which regulates stomatal closure, halts growth, and induces transcriptional changes that enable the plant to adapt to water stress [22]. The activation of these genes is associated with damage mitigation and increased stress tolerance. In this context, osmoprotective compounds such as raffinose, mannitol, fructans, glycine betaine, and proline play essential roles in maintaining osmotic balance, controlling water flow, preserving membrane integrity, and facilitating the synthesis of proteins that deactivate reactive oxygen species and degrade defective proteins. Together, these mechanisms help maintain the integrity of the plant’s photosynthetic and respiratory systems [21,23,24]. Therefore, species such as wild passion fruit, which exhibit high resistance to water deficits, can perform well even under rainfed conditions, provided they are properly managed.

Understanding the interaction between ripening stages and cultivation systems is essential for the efficient management of wild passion fruit in the Brazilian semi-arid region. For this reason, this study aimed to evaluate the influence of cultivation systems (irrigated and rainfed) and different ripening stages on the physical and post-harvest characteristics of wild passion fruit during the second production cycle.

## 2. Results

**Figure 1:**
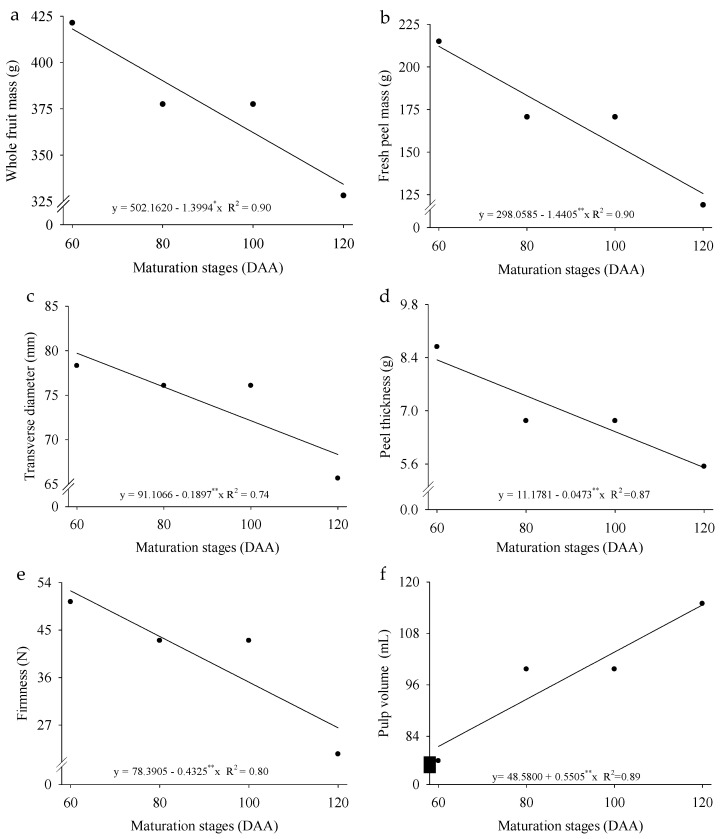
Whole fruit mass (**a**), fresh peel mass (**b**), transverse diameter (**c**), peel thickness (**d**), peel firmness (**e**), and pulp volume (**f**) of *Passiflora cincinnata* ‘BRS Sertão Forte’ under irrigated and rainfed cultivation systems as a function of days after anthesis (DAA). The lines represent the linear regression models adjusted to the means. Asterisks (** and *) in the equations indicate that the model coefficients are significant at *p* ≤ 0.05 and *p* ≥ 0.01, respectively, by the F-test.

The physical characteristics of *P. cincinnata* were independently influenced by ripening stages and cultivation systems (Figure 1a–e). As fruit development progressed, there was a significant reduction in total fruit mass (27.86%), fresh peel mass (48.32%), transverse diameter (20.82%), peel thickness (42.31%), and firmness (55.17%). On the other hand, pulp volume showed a linear increase of 0.5505 mL per day, representing a growth of 1.13% (Figure 1f).

**Figure 2:**
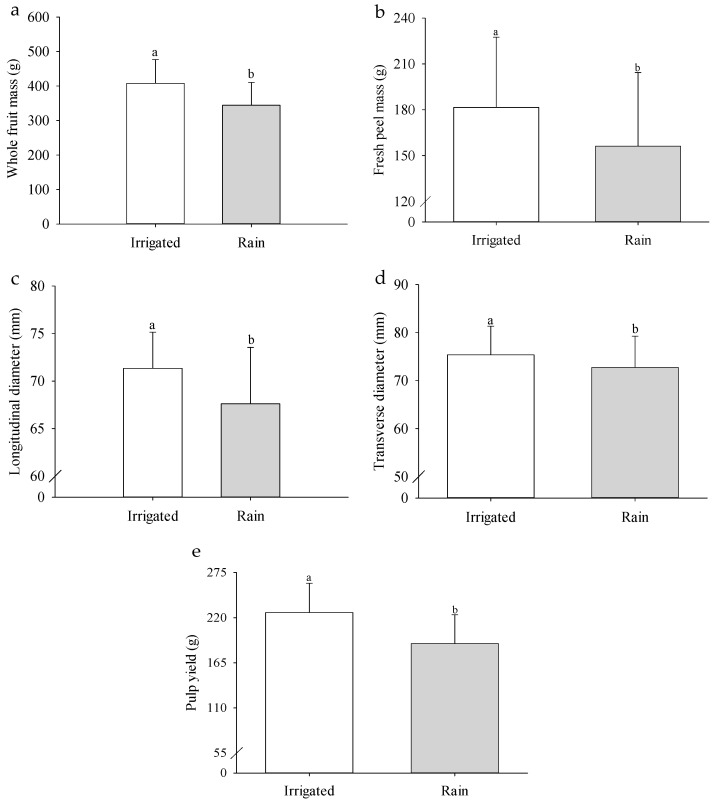
Whole fruit mass (**a**), fresh peel mass (**b**), longitudinal diameter (**c**), transverse diameter (**d**) and fruit yield (**e**) of *Passiflora cincinnata* ‘BRS Sertão Forte’ under irrigated and rainfed cultivation systems as a function of days after anthesis. Means followed by different letters (a,b) indicate a significant difference between cultivation systems according to the Tukey test (*p* ≤ 0.05). Bars represent the standard error of the mean.

When comparing the overall effect of the two cultivation systems, the rainfed treatment, characterized by water restriction, resulted in significant reductions in several fruit parameters compared to the irrigated system. Specifically, reductions were observed in total fruit mass (15.50%), fresh peel mass (13.94%), longitudinal diameter (5.23%), transverse diameter (3.49%), and fruit yield (16.76%) of the *P. cincinnata* cultivar ‘BRS Sertão Forte’ (Figure 2a–e).

**Figure 3:**
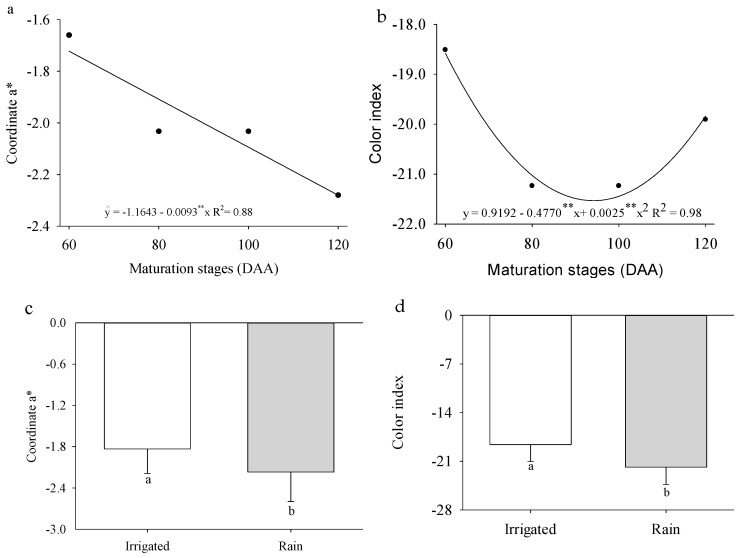
Coordinate *a* and (**a**) color index (**b**) of *Passiflora cincinnata* ‘BRS Sertão Forte’ as a function of days after anthesis; coordinate a (**c**) and color index (**d**) of *Passiflora cincinnata* ‘BRS Sertão Forte’ under irrigated and rainfed cultivation systems. For graphs (**a**,**b**), the curves represent the regression models adjusted to the means; asterisks (** and *) denote significance for the model coefficients at *p* ≤ 0.05 and *p* ≥ 0.01, respectively. For graphs (**c**,**d**), means followed by different letters indicate a significant difference by the Tukey test (*p* ≤ 0.05).

The coordinate *a* and color index were independently influenced by the evaluated factors (Figure 3a–d). The coordinate a (green) showed an 89.46% reduction as the fruits developed, while the color index reached its highest value at 95 DAA. In the rainfed cultivation system, there were reductions of 15.44% and 14.82% in the coordinate a and fruit color index, respectively.

**Figure 4:**
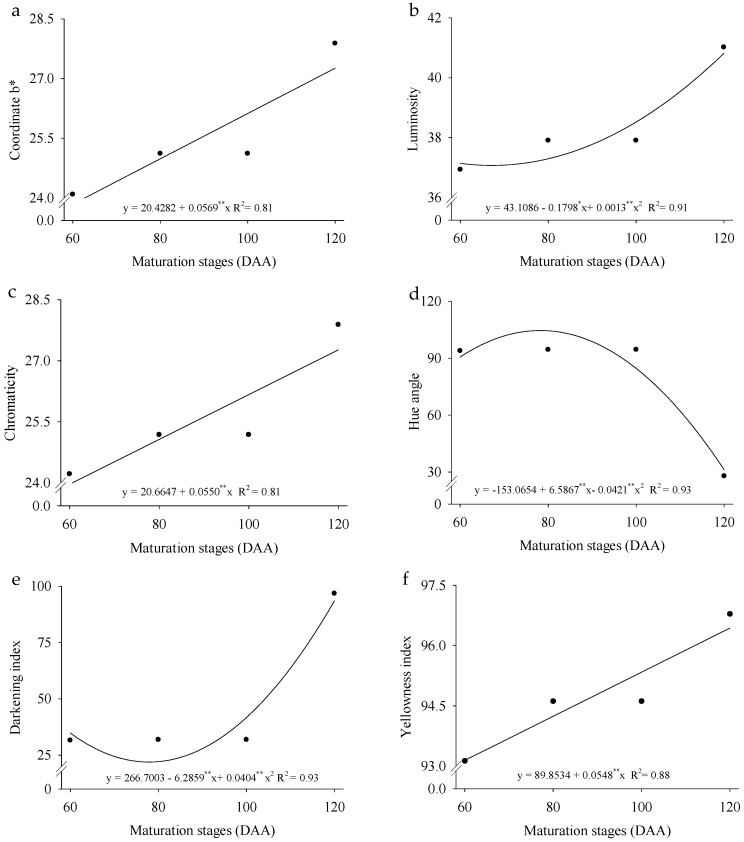
Coordinate *b* (**a**), luminosity (**b**), chroma (**c**), Hue angle (**d**), darkening index (**e**), and yellowness index (**f**) of *Passiflora cincinnata* ‘BRS Sertão Forte’ under irrigated and rainfed cultivation systems as a function of days after anthesis. The curves represent the regression models (linear or quadratic) adjusted to the means. Asterisks (** and *) in the equations indicate that the model coefficients are significant at *p* ≤ 0.05 and *p* ≥ 0.01, respectively, by the F-test.

The remaining color variables were significantly influenced by the fruit ripening stages, with the coordinate b (yellow), chroma, and yellowness index increasing by 33.14, 31.67, and 6.83%, respectively, in harvests conducted between 60 and 120 DAA (Figure 4a–f). Luminosity (L) and the browning index reached their lowest estimated values at 69 and 78 DAA, corresponding to 36.89 and 22.19, respectively. Meanwhile, the Hue angle showed a maximum estimated value of 104.56 at 78 DAA, followed by a 70.2% reduction when the fruits were harvested at 120 DAA, reaching a final Hue angle value of 31.09. These results highlight the dynamic changes in fruit color attributes throughout ripening and under different cultivation systems, reflecting their influence on fruit quality and visual appeal.

**Figure 5:**
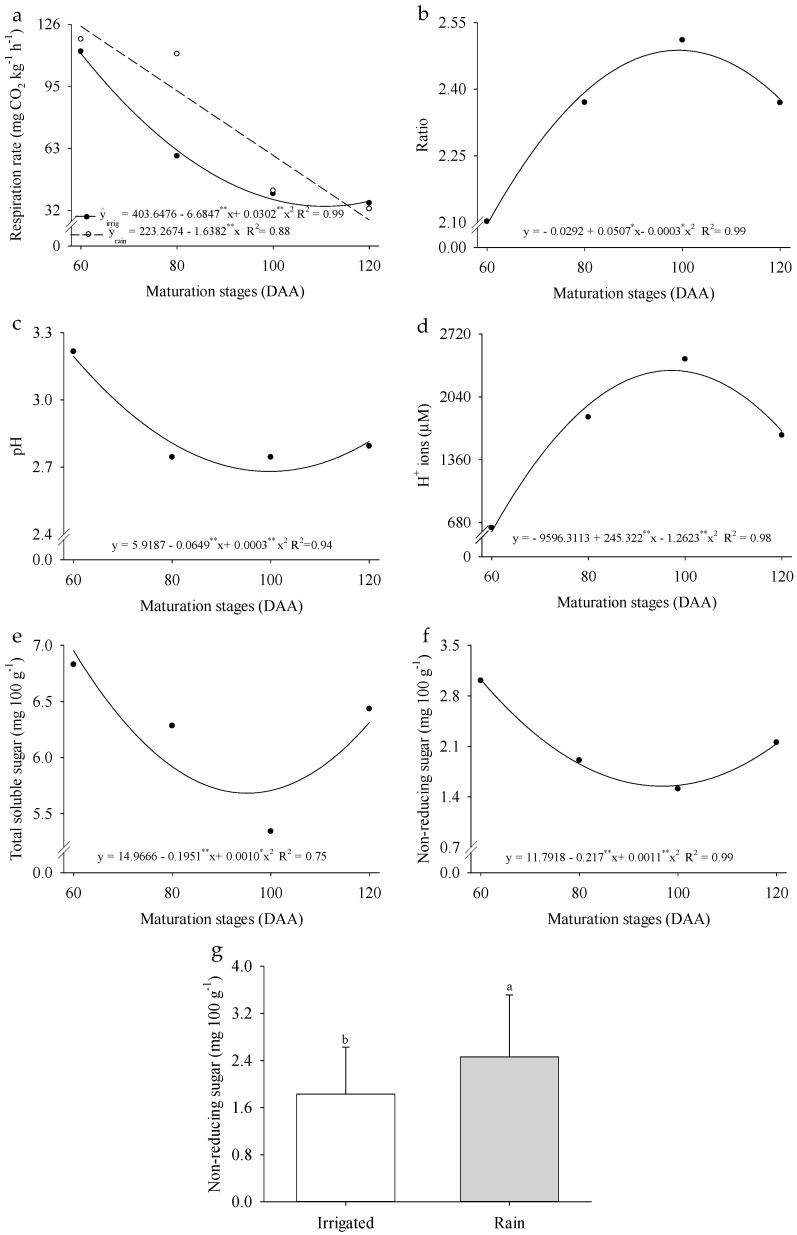
Respiration rate (**a**), SS/TA ratio (**b**), pH (**c**), H^+^ ions (**d**), total soluble sugar (**e**), and non-reducing sugar (**f**,**g**) of *Passiflora cincinnata* ‘BRS Sertão Forte’ under irrigated and rainfed cultivation systems as a function of days after anthesis. For graphs (**a**–**f**), the curves represent the regression models adjusted to the means; asterisks (* and **) denote significance for the model coefficients at *p* ≤ 0.05 and *p* ≥ 0.01, respectively. For graph (**g**), means followed by different letters indicate a significant difference by the Tukey test (*p* ≤ 0.05).

There was an interaction between the evaluated factors for the fruit respiration rate (Figure 5a). In the rainfed cultivation system, the respiration rate of *P. cincinnata* fruits decreased linearly by 73.37% throughout their development, while in irrigated cultivation, the minimum estimated respiration rate was 33.74 mg CO_2_ kg^−1^ h^−1^ at 110 DAA. However, the SS/TA ratio, pH, H^+^ ion concentration, and total soluble sugars differed only across the different fruit ripening stages (Figure 5b–e). The SS/TA ratio reached its maximum value (2.11) at 85 DAA, while pH had its minimum value (2.41) at 108 DAA. The H^+^ ion concentration peaked at 2325.91 µM at 98 DAA, and total soluble sugars recorded their lowest levels (5.86 g 100 g^−1^) at 88 DAA. Non-reducing sugars, however, were influenced by the cultivation system, showing a 26.01% increase under rainfed conditions compared to irrigated cultivation (Figure 5f). These results highlight the distinct physiological and biochemical responses of *P. cincinnata* fruits under different cultivation systems and ripening stages, emphasizing their impact on fruit quality parameters.

**Figure 6:**
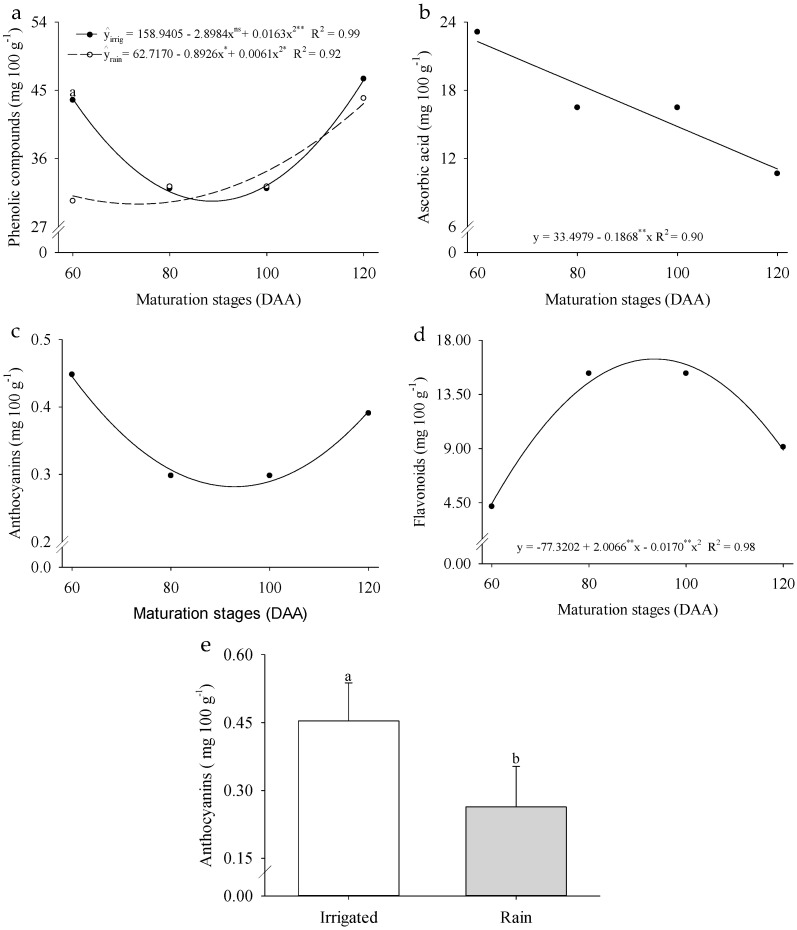
Phenolic compounds (**a**), ascorbic acid (**b**), anthocyanins (**c**,**e**), and flavonoids (**d**) of *Passiflora cincinnata* ‘BRS Sertão Forte’ under irrigated and rainfed cultivation systems at different ripening stages. For graphs (**a**–**d**), the curves represent the regression models adjusted to the means; asterisks (** and *) denote significance for the model coefficients at *p* ≤ 0.05 and *p* ≥ 0.01, respectively. For graph (**e**), means followed by different letters indicate a significant difference by the Tukey test (*p* ≤ 0.05).

Regarding the phenolic compounds in wild passion fruit, a significant interaction was observed between the evaluated factors, with minimum values of 30.10 and 30.06 mg 100 g^−1^ at 89 and 73 DAA under irrigated and rainfed cultivation systems, respectively (Figure 6a). Ascorbic acid content decreased by 55.76% as fruit development progressed from 60 to 120 DAA (Figure 6b). Anthocyanin levels differed between cultivation systems, with the rainfed system reducing this antioxidant compound by 41.90% (Figure 6c). The highest flavonoid content (16.76 mg 100 g^−1^) was observed at 94 DAA (Figure 6d). These findings underscore the influence of cultivation systems and ripening stages on the biochemical composition of *P. cincinnata* fruits, highlighting their impact on the dynamics of antioxidant compounds.

**Figure 7:**
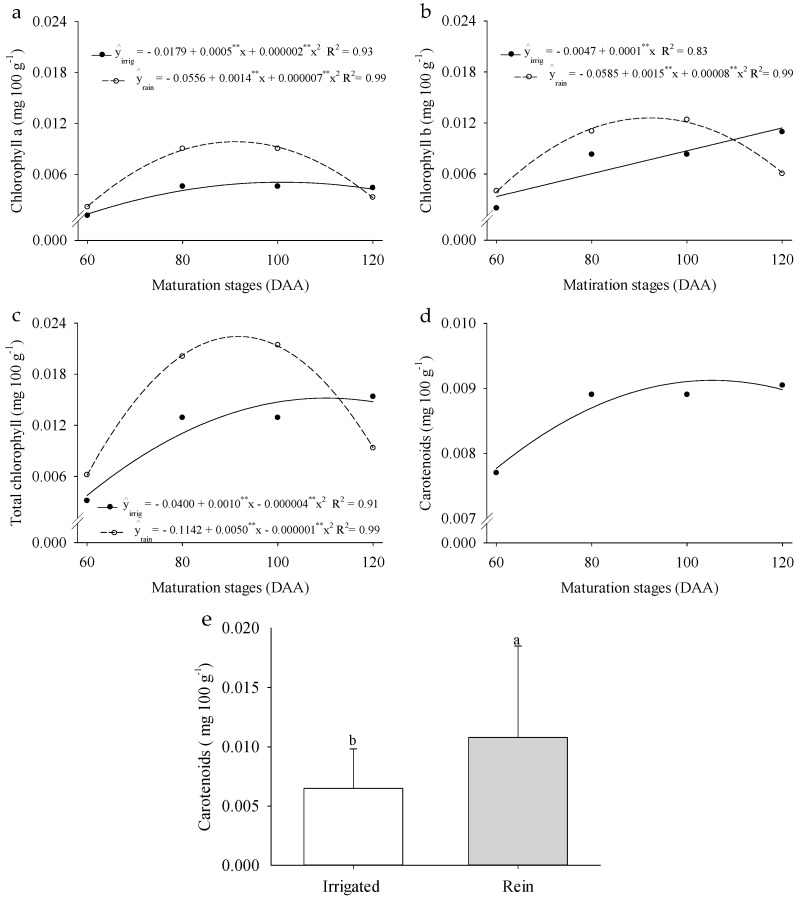
Chlorophyll a (**a**), chlorophyll b (**b**), total chlorophyll (**c**), and carotenoids (**d**,**e**) of *Passiflora cincinnata* ‘BRS Sertão Forte’ under irrigated and rainfed cultivation systems at different ripening stages. For graphs (**a**–**d**), the curves represent the regression models adjusted to the means; asterisks (**) denote significance for the model coefficients at *p* ≤ 0.05. For graph (**e**), means followed by different letters indicate a significant difference by the Tukey test (*p* ≤ 0.05).

For chlorophylls a and b and total chlorophyll, a significant interaction was also observed between cultivation systems and different fruit ripening stages (Figure 7a–c). In the rainfed cultivation system, the maximum chlorophyll content was recorded between 80 and 100 DAA. However, in the irrigated system, chlorophyll a peaked after 100 DAA, while chlorophyll b and total chlorophylls reached their maximum levels at 120 DAA. Carotenoid content increased by 39.89% when fruits were cultivated under the rainfed system (Figure 7d). These results highlight the distinct accumulation patterns of photosynthetic pigments in *P. cincinnata* fruits under different cultivation systems and ripening stages, reflecting their physiological adaptations to water availability.

**Figure 8:**
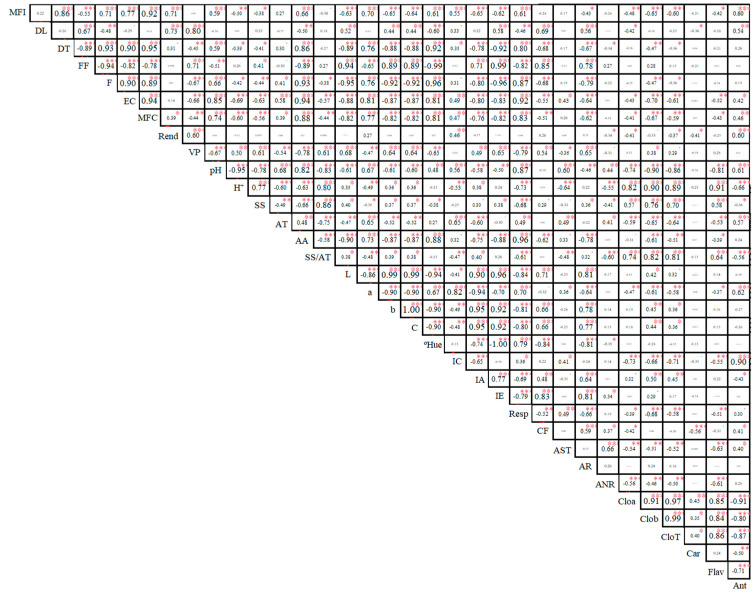
Pearson’s correlation between physical, chemical, and bioactive compound characteristics of *Passiflora cincinnata* ‘BRS Sertão Forte’ fruits under irrigated and rainfed cultivation systems at different ripening stages. Respiration = Resp, fresh peel mass = FPM, peel thickness = PT, whole fruit mass = WFM, transverse diameter = TD, longitudinal diameter = LD, firmness = F, fruit shape = FS, yield = Y coordinate a = a, coordinate b = b, luminosity = L, chroma = C, color index = CI, browning index = BI, yellowness index = YI, Hue angle = Hue, hydrogen potential = pH, titratable acidity = TA, soluble solids = SS, soluble solids/titratable acidity ratio = SS/TA, H^+^ ions = H^+^, ascorbic acid = AA, chlorophyll a = Cloa, chlorophyll b = Clob, total chlorophyll = CloT, carotenoids = Car, flavonoids = Flav, anthocyanins = Ant, total soluble sugars = TSS, reducing sugars = RS, non-reducing sugars = NRS. *, **, *** Significantly different at 5%, 1%, and 0.1%, respectively, by the *t*-test.

The variables whole fruit mass (WFM), peel fresh mass (PFM), longitudinal diameter (LD), pulp volume (PV), transverse diameter (TD), peel firmness (PF), peel thickness (PT), ascorbic acid (AA), Hue angle (Hue), respiration (Resp), fruit shape (FS), luminosity (L), coordinate b (b), chromaticity (C), browning index (BI), phenolic compounds (PC), hydrogen potential (pH), H^+^ concentration (H^+^), soluble solids/titratable acidity ratio (SS/TA), chlorophyll a (Cloa), chlorophyll b (Clob), total chlorophyll (CloT), flavonoids (Flav), and soluble solids (SS) were positively correlated according to Pearson’s correlation analysis (Figure 8).

The variables soluble solids/titratable acidity ratio (SS/TA), chlorophyll b (Clob), total chlorophyll (CloT), luminosity (L), coordinate b (b), chromaticity (C), yellowness index (YI), browning index (BI), reducing sugars (RS), coordinate a (a), color index (CI), anthocyanins (Ant), phenolic compounds (PC), chlorophyll a (Cloa), and flavonoids (Flav) were positively correlated (Figure 8).

In contrast, the transverse diameter (TD), fruit shape (FS), peel firmness (PF), peel thickness (PT), ascorbic acid (AA), luminosity (L), coordinate a (a), coordinate b (b), chromaticity (C), Hue angle (Hue), browning index (BI), respiration (Resp), hydrogen potential (pH), H^+^ concentration (H^+^), chlorophyll b (Clob), and total chlorophyll (CloT) showed negative correlations. Additionally, chlorophylls a and b and total chlorophyll (Cloa, Clob, CloT) were negatively correlated with anthocyanins (Figure 8).

**Figure 9:**
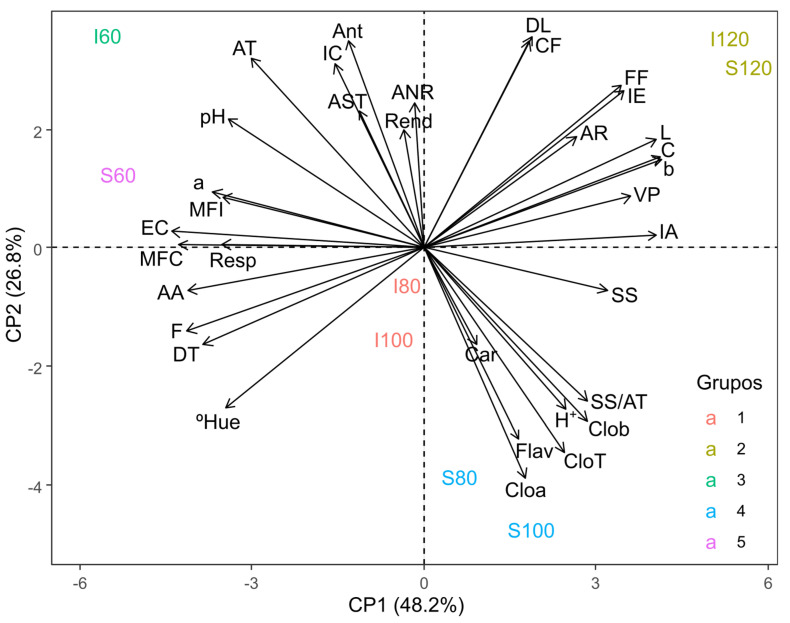
Principal component analysis (PCA) of physical, chemical, and bioactive compound characteristics of *Passiflora cincinnata* ‘BRS Sertão Forte’ fruits under irrigated and rainfed cultivation systems at different ripening stages. Respiration = Resp, fresh peel mass = FPM, peel thickness = PT, whole fruit mass = WFM, transverse diameter = TD, longitudinal diameter = LD, firmness = F, fruit shape = FS, yield = Y, pulp volume = PV, coordinate a = a, coordinate b = b, luminosity = L, chroma = C, color index = CI, yellowness index = YI, Hue angle = ºHue, hydrogen potential = pH, titratable acidity = TA, soluble solids = SS, soluble solids/titratable acidity ratio = SS/TA, H^+^ ions = H^+^, ascorbic acid = AA, chlorophyll a = Cloa, chlorophyll b = Clob, total chlorophyll = CloT, carotenoids = Car, flavonoids = Flav, anthocyanins = Ant, total soluble sugars = TSS, reducing sugars = RS, non-reducing sugars = NRS.

The first two principal components (PC1 and PC2) explained 75% of the total variance, as shown in Figure 9. Additionally, five distinct groups were formed. Respiration rate (Resp), peel fresh mass (PFM), peel thickness (PT), whole fruit mass (WFM), and the coordinate a (a) showed a stronger association when fruits were harvested at 60 DAA under rainfed cultivation (S60). On the other hand, hydrogen potential (pH), titratable acidity (TA), color index (CI), total soluble sugars (SS), anthocyanins (Ant), yield (Y), and non-reducing sugars (NRS) were higher when fruits were harvested at 60 DAA under irrigated cultivation (I60). Longitudinal diameter (LD), phenolic compounds (PC), fruit shape (FS), browning index (BI), reducing sugars (RS), luminosity (L), chroma (C), the coordinate b (b), pulp volume (PV), and yellowness index (YI) were higher when fruits were harvested at 120 DAA, regardless of the cultivation system (I120 and S120). These findings suggest that at 120 DAA, the fruits are fully mature and are no longer significantly influenced by the cultivation system.

Soluble solids (SS), soluble solids/titratable acidity ratio (SS/TA), chlorophyll b (Clob), H^+^ ions (H^+^), total chlorophyll (CloT), flavonoids (Flav), carotenoids (Car), and chlorophyll a (Cloa) showed a stronger association when fruits were harvested at 80 and 100 days after anthesis (DAA) under rainfed cultivation (S80 and S100). This suggests that fruits experience greater stress during this period, notably intensifying the ripening process through the transformation of primary metabolites into secondary metabolites. Meanwhile, the hue angle (Hue), transverse diameter (TD), firmness (F), and ascorbic acid (AA) were more closely associated with fruits harvested at 80 and 100 DAA under irrigated cultivation (I80 and I100), as shown in Figure 9. This indicates that water availability during this phase ensures complete fruit ripening, supporting the development of optimal physical and biochemical characteristics.

## 3. Discussion

The fruit ripening stages influenced total fruit mass, fresh peel mass, transverse diameter, peel thickness, and peel firmness in wild passion fruit (*P. cincinnata* ‘BRS Sertão Forte’) (Figure 1a–e). This indicates that, throughout fruit development, there is an accumulation of osmotically active solutes, such as soluble sugars and organic acids (malic and citric acids), which contribute both to fruit growth and cell expansion in fleshy fruits [25]. Under water-restriction conditions, the limitation of these compounds compromises the fruit’s ability to accumulate water. Nevertheless, the accumulation of osmotically active solutes under these conditions favors a reduction in osmotic potential, helping to maintain cellular turgor pressure [26].

**Figure 10:**
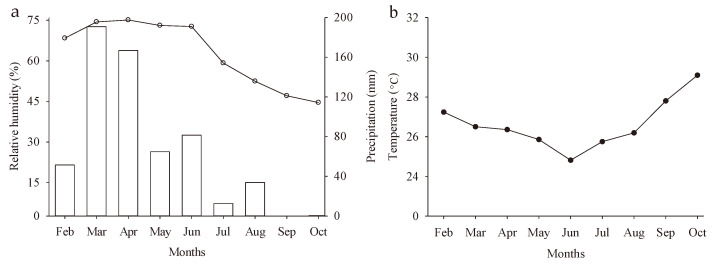
Precipitation and relative humidity of the air (**a**) and air temperature (**b**) during crop development in the field.

Additionally, genetic factors and edaphoclimatic conditions in which the plant is cultivated also influence growth [27]. Therefore, reductions in humidity and precipitation, combined with increased temperatures during the months in which the experiment was conducted, help explain the values observed in this study (Figure 10a,b).

In wild passion fruit (*P. cincinnata)*, increasing the ripening stage leads to a reduction in fruit diameter and mass, [28] a pattern also observed in this study. In yellow passion fruit, starting at 35 DAA, there is a decline in the fresh matter accumulation rate, with no additional growth observed after 60 DAA. However, between 21 and 60 DAA, pulp accumulation occurs, while the peel serves as the primary sink for assimilates until 21 DAA [29]. This behavior is attributed to water loss in the pericarp, which allows the pulp to remain relatively intact despite the reduction in total fruit mass [30].

Regarding the cultivation system, it was observed that *P. cincinnata* plants grown under irrigation produced fruits with greater whole fruit mass, fresh peel mass, longitudinal and transverse diameters, and yield compared to those grown under rainfed conditions (Figure 2a–e). These results are associated with a higher accumulation of photoassimilates, sugars, and carbohydrates, as has been observed in persimmon fruits (‘Giombo’ and ‘Fuyu’) [31], which occurs more efficiently in a well-hydrated environment. Conversely, under rainfed conditions, water limitation hinders the translocation of these metabolites due to interference in xylem transport mechanisms and a reduction in transpiration flow, as verified in coffee [32], resulting in fruits with smaller dimensions.

Wild passion fruit responded positively to drip irrigation, highlighting the importance of proper water management. Regular water supply throughout the crop cycle promotes the establishment of a favorable microclimate for plant development, while also enhancing photosynthesis and transpiration processes [33]. These factors contribute to the superior performance observed in fruits from plants grown under irrigation, reinforcing the critical role of consistent water availability in optimizing fruit quality and yield.

The ripening of wild passion fruit induces structural changes, such as a reduction in peel firmness and thickness, along with an increase in pulp volume throughout fruit development (Figure 1e,f). The decrease in firmness is attributed to modifications in cell wall polysaccharides, primarily pectins, which reduce intercellular adhesion and cause progressive cell wall disintegration during ripening, a mechanism also described for apricot (*Prunus armeniaca*) [34]. Endo- and exo-polygalacturonase enzymes present in passion fruit and papaya, for example, play a key role in pectin solubilization, depolymerization, and rearrangement, contributing to the loss of cell wall cohesion [35]. In wild passion fruit, this loss of firmness and peel thickness also results in the transfer of materials from the peel to the pulp and increased water loss (Figure 1e,f). The observed reduction in fruit weight, despite the increase in pulp volume, may be associated with significant moisture loss and the degradation of structural components in the peel as the fruit ripens. As ripening progresses, the peel becomes thinner and less firm due to cell wall degradation, which reduces its contribution to the total fruit mass.

The increase in pulp volume observed in this study is consistent with the findings of [14], who reported higher pulp yield with seeds in *P. cincinnata* during ripening. These processes reflect metabolic adjustments directed towards the accumulation of reserves in the pulp, which are characteristic of the ripening process.

The coloration of *P. cincinnata* fruits exhibited gradual changes during ripening. The a coordinate indicated a transition from green to yellow (Figure 3a), while the b coordinate reflected a shift from yellow to red (Figure 4a), supported by the values of the Hue angle (Figure 4d) and the yellowness index (Figure 4f). The high coordinate b values suggest a predominance of yellow coloration, resulting from the degradation of chlorophylls and the synthesis of carotenoid pigments, a process also observed in fresh mango (*Mangifera indica*), which is responsible for the yellow and red hues observed during the final stages of ripening [36].

Although the yellow coloration is not yet visible at this stage, as it is masked by the green chlorophyll pigments—indicated by the negative a coordinate value, as also observed in Fino 49 lemons [37]—the fruits tend to exhibit predominantly green coloration during immaturity due to the presence of chlorophyll. As ripening progresses, carotenoids accumulate, imparting a characteristic yellow color, as seen in *Capsicum* ssp. [38]. Ref. [39] observed that, in *Kinnow mandarin,* a citrus fruit exposed to different environmental conditions, carotenoids are the primary pigments responsible for color change during ripening, accumulating mainly in the peel as chlorophyll degrades. Additionally, factors such as moisture content, temperature, and photochemical reactions can influence fruit color by altering the chemical composition of fruit tissues [40].

When comparing color variables across different tangerine cultivars and ripening stages, [41] observed that the a coordinate values during the first harvest period (HT 1) were around −10, indicating a predominantly green coloration at this stage. In citrus fruits, carotenoid content is the primary determinant of yellow coloration, and its increase is directly associated with the ripening process [42]. Consequently, the increase in luminosity (L) and chromaticity (C) values throughout ripening gives the fruit a lighter and brighter appearance [43].

The respiration of *P. cincinnata* fruits was influenced by the interaction between the studied factors (Figure 5a). Respiration is one of the metabolic processes responsible for fruit mass loss due to water loss and sugar consumption, as seen in other passion fruit varieties [44]. Consequently, the decline in soluble sugars is attributed to respiration, where sugars are converted into CO_2_ and H_2_O, a process detailed in studies of sweet cherry (*Prunus avium* L.) [45]. However, the similarity in the behavior of total soluble sugars and non-reducing sugars confirms the increase in the SS/TA ratio, as non-reducing sugars lack free ketone or aldehyde groups and are not readily oxidized, making their relative accumuation more apparent during ripening. The sugar and organic acid content in citrus fruits are important indicators of ripening, serving as essential parameters for determining fruit quality and harvest readiness [46].

The soluble solids content in fruits can vary depending on the cultivar and temperature, as shown in strawberry [47]. Citric acid is the primary organic acid accumulated, with its reserves increasing early in fruit growth and reaching a peak rapidly. This process is strongly influenced by factors such as nutritional conditions and temperature, a process studied in yellow passion fruit [48]. During ripening, high temperatures tend to accelerate the reduction in acid concentration in Valencia oranges [49]. In fruits that do not store starch, such as passion fruit, sugars are synthesized from organic acids [50].

The consumption of organic acids becomes evident at 120 DAA, a period marked by a reduction in the SS/TA ratio and H^+^ ion concentration, while pH and total soluble sugars show a slight increase. In fruits from *P. cincinnata* cultivated under rainfed conditions, an increase in non-reducing sugars was observed, likely resulting from metabolic adjustments that favor the accumulation of organic acids. This metabolite accumulation in plants subjected to water deficit, as described for different passion fruit cultivars, contributes to a reduction in osmotic potential, driven by increased intracellular solute concentrations [51].

Bioactive compounds, particularly phenolic compound levels, were influenced by the interaction between ripening stages and cultivation systems in *P. cincinnata.* These compounds are characterized by a basic structure consisting of an aromatic ring attached to a hydroxyl group (–OH), which can be substituted by other functional groups. The biosynthesis of phenolic compounds occurs predominantly through the phenylpropanoid pathway, also known as the shikimate pathway, which plays a central role in the formation of these metabolites [52].

The increase in phenolic compound levels observed in plants grown under rainfed conditions, especially during fruit ripening, is associated with the activation of the enzyme phenylalanine ammonia-lyase (PAL). This enzyme, often stimulated by various stress factors, plays a key role in the phenylpropanoid pathway, catalyzing the conversion of phenylalanine into cinnamic acid. Cinnamic acid, in turn, serves as a precursor for p-coumaric, caffeic, ferulic, and sinapic acids through a cascade of metabolic reactions [53]. These findings suggest that, under water stress conditions, plants enhance the biosynthesis of phenolic compounds as an adaptive strategy to mitigate the effects of oxidative stress [54].

When evaluating the quantity of phenolic compounds in different passion fruit species (*Passiflora* spp.), *P. edulis* was the only species to present all the studied compounds [55]. In ‘Lane Late’ and ‘Delta’ sweet oranges cultivated under a Mediterranean climate, characterized by 280 mm of rainfall between November and March, phenolic compounds reached maximum values of 363 and 413 mg L^−1^ in December but drastically decreased in January when the fruits were fully ripe [56]. In passion fruit, phenolic compounds measured using the Folin–Ciocalteu method showed values of 365 mg kg^−1^ for *P. edulis* and 476.1 mg kg^−1^ for *P. cincinnata* [57]. These results indicate that phenolic composition is influenced not only by fruit type [46] but also by edaphoclimatic factors, cultivation systems, and storage conditions [58].

The greatest contribution to the total antioxidant activity of wild tropical fruits is not attributed to vitamin C but rather to the phytochemical composition [59]. Ascorbic acid originates from metabolic pathways that utilize sugars derived from the cell wall, as noted in kiwifruit (*Actinidia deliciosa*) [60]. However, the levels of these acids decrease as the fruit ripens, as observed in this study, where ascorbic acid levels declined progressively with fruit ripening (Figure 6b). This phenomenon may be associated with the cultivar’s genetic makeup and environmental factors, such as light exposure during plant and fruit growth, which directly influence the biosynthesis of ascorbic acid [61].

The polyphenol content in fruits and vegetables is primarily determined by genetic factors but can be altered by oxidative reactions caused by biotic and abiotic stress, which impacts the quality of yellow passion fruit, for example, during storage, including temperature, oxygen, and post-harvest conditions [62]. Anthocyanins and flavonoids are responsible for colors ranging from bright red to violet and from white to light yellow, respectively [63,64]. Although anthocyanin levels (Figure 6c) were higher when plants were grown under irrigation, these values were not sufficient to influence the juice coloration of *P. cincinnata*.

The concentrations of chlorophyll a and b and total chlorophyll were influenced by the studied factors, while carotenoid levels were higher under rainfed cultivation conditions. Chlorophylls and carotenoids are essential pigments in plants, playing crucial roles in photosynthetic processes [65]. Additionally, these compounds help protect plants against excessive radiation and oxidative stress [66]. In citrus fruits, chlorophyll levels function as protective barriers against the side effects of heat [67,68]. Similarly, carotenoids contribute to protein stabilization during fruit growth and development, as investigated in *Coffea canephora* and *Coffea arabica* [69].

Through principal component analysis (Figure 9), it was observed that the physical characteristics of *P. cincinnata* fruits are negatively influenced by the rainfed cultivation system at 60 DAA. This behavior can be attributed to water stress experienced by the fruits during the development stage, which causes a reduction in weight due to the deceleration of growth rates during the expansion phase. This effect is directly related to the decrease in cellular turgor pressure, which is essential for proper fruit growth [70].

On the other hand, fruits from plants grown under an irrigated cultivation system at 60 DAA maintained their quality, which can be attributed to the interdependence between the processes of development and ripening, in which one phase is not entirely completed before the next begins [71]. According to the authors, during the ripening, increased respiratory rates and ethylene production required less degradation of organic compounds, to support biological processes such as growth, nutrient uptake, and photoassimilate transport. This is because an adequate water supply ensured the physiological balance necessary to maintain fruit quality.

However, at 80 and 100 DAA, when fruits were cultivated under a rainfed system, the stress caused by increased temperature, decreased humidity, and reduced precipitation (Figure 10) led to an exponential increase in the respiration rate [72], resulting in a decline in fruit quality and shelf life. In contrast, fruits harvested during the same period but grown under an irrigated system experienced less physiological stress. The intense ripening process, supported by an adequate water supply, ensured proper fruit nutrition and minimized the adverse effects on fruit quality [71].

However, fruits harvested at 120 DAA were not significantly influenced by the cultivation system, indicating that the fruits had reached full ripening. At this stage, several changes occur, including chlorophyll degradation, loss of turgor, pectin solubilization, tissue softening, and increased synthesis of volatile compounds. Additionally, an increase in soluble solids content and a reduction in organic acids, including ascorbic acid and phenolic compounds, are observed [71]. It is important to emphasize that determining the optimal harvest time and cultivation system for wild passion fruit should consider the producer’s specific objectives regarding the desired fruit characteristics.

## 4. Materials and Methods

### 4.1. Experimental Place

The experiment was conducted from February to October 2022 under field conditions at the Rolando Enrique Rivas Castellón Experimental Farm, affiliated with the Center for Agro-Food Science and Technology (CCTA) of the Federal University of Campina Grande (UFCG), located in the municipality of São Domingos, PB (06°48′50″ S and 37°56′31″ W, altitude of 190 m). The region has a BSh (hot and dry) climate, according to the Köpper classification [73], characteristic of semi-arid areas. Data on precipitation, temperature, and relative humidity during the experimental period were obtained from the São Gonçalo meteorological station, available on the website of the National Institute of Meteorology (INMET). The monthly averages during the experiment were 66.89 mm of precipitation, 26.62 °C temperature, and 66.99% relative humidity of the air (Figure 10a,b).

### 4.2. Experimental Structure and Design

The experimental design was a randomized block design in a 2 × 4 factorial scheme, corresponding to two cultivation systems for wild passion fruit (irrigated and rainfed) and four fruit ripening stages (60, 80, 100, and 120 DAA—days after anthesis), with five replications and eight plants per plot.

### 4.3. Experimental Procedures

The seeds of the commercial wild passion fruit cultivar ‘BRS Sertão Forte’ were provided by Embrapa Cerrados (Brasília, Brazil). This cultivar was developed through intraspecific crosses between the progenies CPEF2220 and CBAF2334, derived from *P. cincinnata* populations and accessions from the Germplasm Bank and the Passion Fruit Breeding Program of Embrapa Cerrado. Soil fertility analysis in the experimental area was performed at depths of 0–20 cm and 20–40 cm, as well as on the cattle manure used (Table 1). The substrate for sowing was composed of soil, cured cattle manure, and washed sand in a 3:1:1 ratio (*v*/*v*). Fertilization consisted of applying 1 kg of P_2_O_5_ (single superphosphate) and 0.2 kg of micronutrients (Dripsolmicro).

The seedlings were grown in 5 dm^3^ polyethylene bags, using one seed per bag, and kept in a greenhouse. Weed control and irrigation were performed manually. In the experimental area, planting holes measured 40 × 40 × 40 cm. Seedling transplantation occurred 69 days after sowing, when the plants reached a height of 1 m. The irrigation system used was drip irrigation, using emitters with a flow rate of 20 L h^−1^ per plant. The irrigation depth applied, following the methodology for sour passion fruit described by [74], was 16.8 L per plant per day (7.5 mm day^−1^).

The crop was managed using a trellis system with No. 14 smooth wire, spaced 2.5 m between rows and 3 m between plants. The plants were trained until they extended 10 cm above the trellis, at which point the apical bud was pruned to induce the growth of secondary branches. Two secondary branches were selected and guided, one to each side, until they reached 1.5 m. When the secondary branches reached 1.6 m, pruning was performed to stimulate the formation of tertiary branches, which formed the productive curtain. Throughout the experiment, tendrils and unwanted branches were removed weekly to improve crop performance.

**Figure 11:**
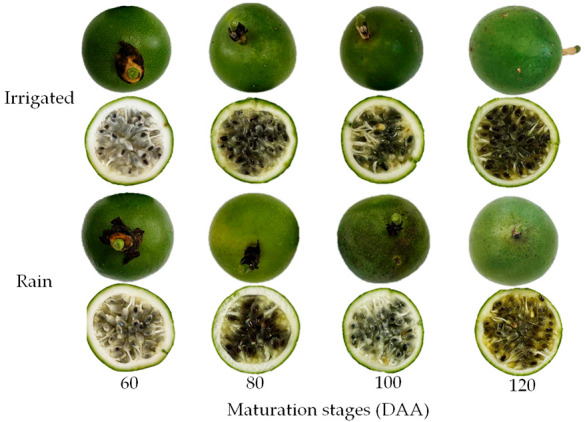
*Passiflora cincinnata* fruits harvested at different ripening stages (60, 80, 100, and 120 DAA) under irrigated and rainfed cultivation conditions.

On the day of anthesis, all flowers were properly marked. The experiment was laid out in 10 rows, each containing 8 plants. For the evaluations, the 6 central plants of each row were selected, disregarding the two end plants to avoid border effects. Harvesting was conducted randomly by collecting six fruits per treatment, totaling 60 fruits for analysis. Flowering began at 97 DAS (days after sowing) and extended until 152 DAS. The flowers opened around 6 a.m. and remained open throughout the day. Pollination was performed manually, and each *P. cincinnata* flower was identified using wool threads tied to the petiole of each properly labeled flower. Fruits were harvested according to the previously established ripening stages (60, 80, 100, and 120 DAA), based on uniformity and appropriate phytosanitary conditions (Figure 11). Harvesting was carried out according to the ripening stages using pruning shears, and the fruits were then placed in Styrofoam boxes with ice. Immediately after harvest, they were transported to the Laboratory of Chemistry, Biochemistry, and Food Analysis at the Universidade Federal de Campina Grande (UFCG), Pombal Campus, where physical and physicochemical analyses were performed.

### 4.4. Variables Analyzed

#### 4.4.1. Respiratory Rate

For the respiratory rate analysis, a sample of six fruits, selected from the total harvested fruits of each replicate, was used. The fruits were weighed using a precision digital scale (model M214-AiH, BEL Engineering, Monza, Italy) and placed in 1.0 L polyethylene containers for six hours, sealed with lids and an added a silicone film to prevent gas exchange with the external environment. Inside each container, a vessel containing 12 mL of 0.5 M NaOH was inserted to fix the CO_2_ produced during respiration. After six hours, the NaOH solution was treated with three drops of phenolphthalein and 10 mL of 0.2 M BaCl_2_ and then titrated with 0.1 M hydrochloric acid. The respiration rate was expressed in mg CO_2_ kg^−1^ h^−1^ following the method described by [75], with adaptations by [76].

#### 4.4.2. Physical Analysis of the Fruit

For the physical analysis, a sample of six fruits per replicate was used. From these, the pulp with seeds was extracted from the wild passion fruit and filtered through a 1 mm polyester sieve. The fresh mass of the whole fruit (including pulp and seeds) and peel as well as the volume of seedless pulp were evaluated, along with the transverse and longitudinal diameters of the whole fruit and peel thickness. Fruit shape was determined by the ratio between the longitudinal and transverse diameters [77]. Peel firmness was measured at the equatorial region on two opposite sides of the fruit using a penetrometer of 3 mm (model PCE-PTR 200, PCE Instruments, Meschede, Alemanha), with results expressed in Newtons (N).

#### 4.4.3. Colorimetry CIE/Lab (L*, a*, and b*)

For each of the six fruits per replicate, colorimetry readings were performed using a Minolta CR-300 colorimeter, with a D65 light source and an 8 mm aperture, based on three color variables: Luminosity (*L*), coordinate *a*, and coordinate *b*. The *L* value represents lightness, ranging from black (*L* = 0) to white (*L* = 100). The *a* value indicates coloration from red (+*a*) to green (−*a*), while the *b* value represents coloration from yellow (+*b*) to blue (−*b*). These parameter values were used to calculate chromaticity (*C*), which indicates the saturation of the analyzed object. The Hue angle (*H°*) is the angle formed between *a* and *b*, indicating the true color of the object [78].

#### 4.4.4. Physicochemical Analysis of the Pulp

For the physicochemical and bioactive compound analyses, the pulp from the six fruits of each replicate was pooled to form a composite sample, from which aliquots were taken for each determination.

#### 4.4.5. pH and Concentration of H^+^ ions

The pH was measured using a bench digital potentiometer (model Digimed DM22, Digimed, São Paulo, Brazil), previously calibrated, with direct readings taken from wild passion fruit pulp samples. The concentration of H+ ions was expressed as the concentration of micromoles (mM) of [H^+^] ions according to the following equation: [H+] = 10-pH [79].

#### 4.4.6. Soluble Solids, Titratable Acidity, and SS/TA Ratio

The wild passion fruit pulps were macerated using a pestle, pipetted, and filtered through a cotton layer. The soluble solids content (SS, °Brix) was determined by direct reading using a digital refractometer (model HI96801, Hanna Instruments, Woonsocket, EUA). Titratable acidity (TA%) was determined by titrating 3 mL of macerated pulp from the composite sample with 47 mL of 0.1 M sodium hydroxide, with the addition of 2 drops of 1% alcoholic phenolphthalein indicator. The SS/TA ratio was expressed as the ratio of soluble solids to titratable acidity [79].

#### 4.4.7. Soluble, Reducing, and Non-Reducing Sugars

From the composite pulp sample, the soluble sugar content (g 100 g^−1^) was determined according to the methodology described by [80]. The reducing sugar content (g 100 g^−1^) was measured using the method described by [81]. These variables were analyzed by spectrophotometry (model SP1105, Shanghai Spectrum Instruments, Shanghai, China) at wavelengths of 620 nm for soluble sugars and 540 nm for reducing sugars. Glucose was used as a reference to establish the standard curve. Non-reducing sugars were calculated as the difference between soluble and reducing sugars.

#### 4.4.8. Analysis of Bioactive Compounds

##### Ascorbic Acid

The ascorbic acid content (mg 100 g^−1^) was determined by titrating 1 mL of the composite pulp sample, completing it with 49 mL of chilled 5% oxalic acid, followed by titration with a 0.2% 2,6-dichlorophenolindophenol solution [82].

##### Total Chlorophyll and Carotenoids

Chlorophyll content (mg 100 g^−1^) was determined using the method proposed by [83]. An extract was prepared by macerating 2 g of the composite pulp sample with 0.2 g of calcium carbonate and 5 mL of 80% acetone in a dark environment. The extract was centrifuged in a refrigerated centrifuge (CT–500R) at 3500 rpm and 10 °C for 10 min. The supernatant was analyzed after 24 h of refrigerated rest using a spectrophotometer (Spectrum SP1105) at wavelengths of 663 nm and 646 nm (chlorophylls *a* and *b*, respectively) and 470 nm (total carotenoids).

##### Flavonoids and Anthocyanins

The contents of flavonoids and anthocyanins (mg 100 g^−1^) were determined using the method described by [84]. An extract was prepared by macerating 0.5 g of the composite pulp sample with 10 mL of ethanol/HCl 1.5 M (85:15, *v*/*v*) in a dark environment, followed by refrigerated rest for 24 h. The extract was centrifuged at 3500 rpm and 10 °C for 10 min. The supernatant was analyzed using a spectrophotometer (Spectrum SP1105) at wavelengths of 374 nm for flavonoids and 535 nm for anthocyanins.

##### Phenolic Compounds

The phenolic compounds (mg 100 g^−1^) were analyzed using the Folin–Ciocalteu method [85]. The extract was prepared with 3 mL of sample and 47 mL of distilled water, followed by 30 min of rest and subsequent filtration. A solution was prepared in test tubes containing 500 µL of the composite pulp sample, 1625 µL of distilled water, and 125 µL of Folin–Ciocalteu reagent, which was shaken (model NI 1107, Nova Instruments, Piracicaba, Brazil) and left to rest for 5 min. Then, 250 µL of 20% sodium carbonate was added, and the tubes were shaken again. The tubes were immersed in a thermostatic water bath (model HM 0105, Hemoquímica, Votuporanga, Brazil) at 40 °C for 30 min. Phenolic compound levels were quantified by spectrophotometer readings (model SP 1105, Shanghai Spectrum Instruments, Xangai, China) at 765 nm, using gallic acid as a reference standard.

### 4.5. Statistical Analysis

The data were subjected to analysis of variance, and the means of the irrigation systems were compared using the F-test (*p* ≤ 0.05), while polynomial regression analysis was applied for the different ripening stages, with Tukey’s test conducted at 5% probability for cultivation systems. The statistical package ExpDes [86] was used in R statistical software (version 4.0.5) [87] for data analysis. Additionally, Pearson’s correlation analysis was performed using the *PerformanceAnalytics* package [88], and principal component analysis (PCA) was conducted to study the interrelationship between variables and factors.

## 5. Conclusions

Wild passion fruits (*Passiflora cincinnata*) should be harvested from 80 DAA onwards, when the SS/TA ratio is optimized, despite reductions in fruit mass, diameter, and peel firmness.

The irrigated system produces fruits with greater total mass, peel mass, and diameter, while the rainfed system favors a higher concentration of non-reducing sugars and carotenoids.

Principal component analysis revealed that, at 60 DAA, the physical characteristics of the fruits were negatively affected under the rainfed cultivation system. In contrast, fruits from the irrigated system maintained better physical quality at the same stage.

At 80 and 100 DAA, fruits grown under the rainfed system exhibited an exponential increase in respiration rate, resulting in reduced quality and post-harvest shelf life. Conversely, fruits harvested during the same period under the irrigated system experienced less stress.

## Figures and Tables

**Table 1:** Chemical attributes of the soil in the experimental area and the manure used for fertilization.

Attributes												
Depth	pH	P	K^+^	Na^+^	H^+^ + Al^3+^	Al^3+^	Ca^2+^	Mg^2+^	SB	CEC	V	OM
(cm)	H_2_O	-- mg dm^−3^--		------------------------------ cmol dm^−3^ ------------------------							%	g dm^−3^
00–20 cm	8.94	46.18	7.89	6.52	0.00	0.00	15.25	24.55	53.91	53.91	100	1.74
20–40 cm	6.78	13.77	0.37	0.91	0.48	0.00	7.41	2.44	11.13	11.61	95.86	1.74
Manure	6.71	16.87	0.51	0.74	0.40	0.00	6.18	1.92	9.35	9.75	95.89	-

SB = sum of bases; CEC = cation exchange capacity; V = base saturation; MO = organic matter.

## Data Availability

All data produced and/or analyzed in this study are included in the manuscript. The corresponding authors are available to provide additional data and materials upon reasonable request.
